# Differential expression of aqueous humor microRNAs in central retinal vein occlusion and its association with matrix metalloproteinases: a pilot study

**DOI:** 10.1038/s41598-022-20834-z

**Published:** 2022-09-30

**Authors:** Eun Hee Hong, Mina Hwang, Hyoseon Yu, Hyun-Hee Park, Heeyoon Cho, Seong-Ho Koh, Yong Un Shin

**Affiliations:** 1 Department of Ophthalmology, Hanyang University College of Medicine, Seoul, Republic of Korea; 2 Department of Neurology, Hanyang University College of Medicine, Seoul, Republic of Korea; 3 Graduate School of Biomedical Science and Engineering, Hanyang University, Seoul, Republic of Korea

**Keywords:** Biomarkers, Retinal diseases, Biomarkers, Pathogenesis, Retina

## Abstract

The aim of this study is to investigate the differential expression of microRNAs (miRNAs) in the aqueous humor (AH) of patients with central retinal vein occlusion (CRVO), and their association with AH matrix metalloproteinase (MMP) activity. Eighteen subjects, including 10 treatment naïve patients with CRVO and 8 control subjects, scheduled for intravitreal injection and cataract surgery, respectively, were included. AH samples were collected at the beginning of the procedure. A microarray composed of 84 miRNAs was performed to identify differentially expressed miRNAs in CRVO AH, which were further analyzed using bioinformatic tools to identify directly related cytokines/proteins. Eight miRNAs (*hsa-mir-16-5p, hsa-mir-142-3p, hsa-mir-19a-3p, hsa-mir-144-3p, hsa-mir-195-5p, hsa-mir-17-5p, hsa-mir-93-5p*, and *hsa-mir-20a-5p*) were significantly downregulated in the CRVO group. Bioinformatic analysis revealed a direct relationship among downregulated miRNAs, CRVO, and the following proteins: MMP-2, MMP-9, tumor necrosis factor, transforming growth factor beta-1, caspase-3, interleukin-6, interferon gamma, and interleukin-1-beta. Activities of MMP-2 and -9 in AH were detected using gelatin zymography, showing significant increase in the CRVO group compared to the control group (*p* < 0.01). This pilot study first revealed that MMP-2 and -9 were directly related to downregulated miRNAs and showed significant increase in activity in AH of patients with CRVO. Therefore, the relevant miRNAs and MMPs in AH could serve as potential biomarkers or therapeutic targets for CRVO.

## Introduction

Central retinal vein occlusion (CRVO), an occlusion of the central retinal vein posterior to the lamina cribrosa of the optic nerve, is one of the main causes of sudden painless vision loss in adults and can lead to severe visual impairment^1^. However, the exact pathogenesis remains unclear. A combination of three systemic changes, known as Virchow’s triad (venous stasis, endothelial damage, and hypercoagulability), contributes to thrombosis and can precipitate CRVO^2,3^. Hypoxia in the retinal tissue and subsequent release of vascular endothelial growth factor (VEGF) and inflammatory mediators lead to further complications, including macular edema, vitreous hemorrhage, and neovascularization, which can cause severe visual impairment^4^. Initial treatment involves intravitreal injection of an anti-VEGF agent. However, some patients respond poorly to this treatment; alternatively, intravitreal injection of steroids could be administered to these patients to reduce the production of several pro-permeability factors^3,5^. Current CRVO treatment strategies emphasize the need for customized treatment according to the disease state.

Circulating microRNAs (miRNAs) have been actively studied as mediators or biomarkers of various diseases^6,7^. The miRNAs are small, non-coding RNAs that regulate gene expression by inhibiting messenger RNA (mRNA) translation and are highly stable in body fluids (in contrast to mRNAs)^8^. Therefore, the role of miRNAs has recently been investigated in ocular diseases, including glaucoma and diabetic retinopathy, using aqueous humor (AH) samples^9,10^. Additionally, miRNAs have potentially significant pathophysiologic relevance in various cardiovascular or thrombotic diseases^7,11^. However, miRNA expression in the AH of patients with CRVO has not been reported.

Matrix metalloproteinases (MMPs) are a family of calcium- and/or zinc-dependent extracellular proteolytic enzymes that degrade various proteins, or remodel the extracellular matrix (ECM) under various physiological and pathological conditions, including ischemic eye diseases^12,13^. Various subfamilies are classified according to function, and MMP-2 and -9 compose the gelatinase subfamily^14^. MMP-2 and -9 are known to be linked to a variety of microvascular endothelial functions, including proliferation, differentiation, and angiogenesis^15^. A limited number of studies have reported the role of MMPs in CRVO. High aqueous or vitreous MMP levels in patients with retinal vein occlusion have been reported^16–18^. However, there are no reports on MMP-related miRNAs that may affect or be affected by MMPs using the AH of patients with CRVO. Thus, the role of MMPs in CRVO remains to be elucidated.

Here, we investigated the differential expression of miRNAs in the AH of patients with CRVO and its association with aqueous humor MMP activity. We also compared the activity of MMP-2 and -9 to confirm the differences between patients with CRVO and control subjects.

## Methods

The protocol of this prospective, cross-sectional study was reviewed and approved by the institutional review board of Hanyang University Guri Hospital (IRB FILE No. 2017-06-026) and adhered to the tenets of the Declaration of Helsinki. All participants provided written informed consent prior to participating in the study.

### Subjects

Eighteen subjects were recruited from the Retina Clinic of the Department of Ophthalmology of Hanyang University Guri Hospital, Gyeonggi-do, South Korea, between March 2018 and March 2021. This included 10 patients with recent-onset-naïve CRVO with macular edema (CRVO group), who were scheduled for intravitreal injection and 8 normal subjects (control group), who were scheduled for cataract surgery. The following inclusion criteria were employed for the CRVO group: treatment naïve CRVO, confirmed by fundus examination and fluorescein angiography (Optos, Dunfermline, Scotland); no history of previous retinal photocoagulation, intravitreal injection of any agent, and pars plana vitrectomy; no history of co-existent retinal pathology; no severe cataract affecting vision; and a sufficiently deep anterior chamber to perform anterior chamber paracentesis without complications. Patients with CRVO were indicated for receiving an intravitreal injection when severe macular edema with visual loss was present: optical coherence tomography (OCT) central subfield thickness≥ 300 µm, and visual acuity ≤ 20/40. Patients with ischemic CRVO were excluded because of the homogeneity within the patient group^19^. The inclusion criteria of the control group were: no history of ocular diseases other than cataracts; no prior intraocular surgery; and no systemic diseases other than hypertension. Subjects in both groups were excluded if they had a history of intraocular or systemic inflammatory diseases, cancer, autoimmune diseases, liver and kidney dysfunction, ocular trauma, or intraocular surgery (except for cataract surgery more than 6 months ago in the CRVO group), or if the amount of AH sample collected was not sufficient or was deemed inappropriate for analysis. All participants underwent standard ophthalmologic examination, including best-corrected visual acuity, intraocular pressure, slit lamp biomicroscopy, OCT (Swept Source OCT, Topcon DRI OCT-1 Atlantis; Topcon, Inc., Tokyo, Japan), and Optos ultra-wide fundus photography. Patients with CRVO also underwent fluorescein angiography.

### Sample preparation

Aqueous humor samples were collected at the beginning of the cataract surgery or intravitreal injection procedure, using the following procedure: after sterile surgical draping was performed, a sterile eyelid speculum was placed and 1–2 drops of 0.5% proparacaine hydrochloride (Paracaine, Alcon, Ft. Worth, TX, USA) and 5% povidone iodine were instilled immediately before surgery or injection. Anterior chamber paracentesis was performed using a 30-gauge needle mounted on a 1 mL syringe, through a puncture at the peripheral cornea under a surgical microscope. Aqueous humor sample volumes ranged from 30 to 150 µL. The samples were transferred to microtubes and immediately stored at − 80 °C.

### Extraction of miRNA and cDNA synthesis

The miRNAs obtained from AH samples were isolated using an miRNeasy serum/plasma kit (Qiagen, Hilden, Germany), according to the manufacturer’s instructions. All samples were added to miRNeasy serum/plasma spike-in control (Qiagen) as an internal control for normalization at 5.6 × 10^8^ copies. Using a miScript nucleic mix, miScript reverse transcriptase mix, miScript Hispec buffer, and RNase-free water in the miScript II RT kit (Qiagen), 50 ng RNA samples were reverse-transcribed to synthesize cDNA. The mixtures were incubated for 60 min at 37 °C and for 5 min at 95 °C, followed by dilution with RNase-free water.

To execute the miRNA PCR array, the synthesized cDNA was pre-amplified using HotStarTaq DNA polymerase, miScript PreAMP universal primer, miScript PreAMP Buffer, miScript PreAMP primer mix, and RNase-free water using the miScript PreAMP PCR kit (Qiagen). The mixture was activated by heating for 15 min at 95 °C. Samples were then reacted for 18 cycles of 30 s at 94 °C and 3 min at 60 °C for denaturation and annealing/extension. The pre-amplified cDNA was then diluted in RNase-free water.

### Real-time PCR and miRNA PCR arrays

Three miScript primers were used for the pre-amplified cDNA quality test. The miR-16 miScript primer was used to determine the optimal dilution factor of pre-amplified cDNA, which avoids overloading or over-diluting the cDNA for miRNA PCR arrays. The *C. elegans* miR-39 miScript primer was used to measure the miRNeasy serum/plasma spike-in control to determine recovery from the samples. The miRTC miScript primer was used to assess the reverse-transcription efficiency. Each miScript primer was mixed with miScript universal primer, QuantiTect SYBR Green PCR master mix, RNase-free water in the miScript SYBR green PCR kit (Qiagen), and pre-amplified cDNA. The mixtures were activated by heating for 15 min at 95 °C and reacted for 45 cycles of 15 s at 94 °C, 30 s at 55 °C, and 30 s at 70 °C. Fluorescence data were collected and analyzed.

For miScript miRNA PCR arrays, a 96-well format of Human miFinder (MIHS-001ZC, Qiagen) was used with the miScript SYBR Green PCR kit. The Human miFinder miRNA PCR array profiles the expression of the 84 most abundantly expressed and best-characterized miRNAs in the latest version of miRBase (www.miRBase.org). These were based on miRBase V10. Three miScript primers were used for calibration according to the above cycler conditions. RNU6-6p in Human miFinder was simultaneously used to normalize the miRNA PCR array results. A list of 84 genes from Human miFinder (96-well format), excluding the 12 controls, is provided in Supplementary Figure S1. The results were analyzed using the online Qiagen Data Analysis Center (http://www.qiagen.com/) and displayed as a heatmap and clustergram.

### Bioinformatics

The relationship between miRNA genes and CRVO was constructed using the Laverne: Bioinformatics Tool from Novus Biologicals (http://www.novusbio.com/explorer). The lines of the bioinformatic images were based on the supporting evidence.

### Gelatin zymography

MMP-2 and -9 activity in the AH were detected using gelatin zymography. Briefly, AH samples were added to a lysis solution [1 mM sodium fluoride (Sigma, St. Louis, MO, USA), 1 mM sodium orthovanadate (Sigma), 0.5% protease inhibitor cocktail 1 × (GenDEPOT, Katy, TX, USA), and 1 mM phenylmethylsulfonyl fluoride (Sigma)] without EDTA and incubated on ice for 30 min. The mixtures were then sonicated using Sonoplus (Bandelin Electronics, Berlin, Germany) and incubated on ice for 30 min. The lysates were centrifuged at 16,200 × *g* for 15 min at 4 °C and the supernatant was transferred to a new microtube. The total protein concentration of the supernatant was calculated using a mixture of copper (II) sulfate solution (Sigma) and bicinchoninic acid solution (Sigma). Equal amounts of protein (13 µg) were dissolved in non-reducing sodium dodecyl sulfate (SDS) sample buffer and resolved on a 7.5% acrylamide gel containing 0.1% gelatin without boiling. After electrophoresis, the gels were soaked in washing buffer [2.5% Triton X-100 (Sigma), 5 mM calcium chloride (CaCl_2_, Sigma), 1 µM zinc chloride (ZnCl_2_, Sigma), and 50 mM Tris–HCl, pH 7.5 (Bio-Rad, Hercules, CA, USA) in distilled water] twice for 30 min to remove SDS from the gel and then rinsed with incubation buffer (1% Triton X-100, 5 mM CaCl_2_, 1 µM ZnCl_2_, and 50 mM Tris–HCl, pH 7.5 in distilled water) for 10 min at 37 °C. Gels were then placed in fresh incubation buffer and incubated for 24 h at 37 °C with agitation to induce the gelatinase reaction. After incubating for 24 h, gels were stained with Coomassie blue for 1 h and rinsed with distilled water. Stained gels were then incubated with destaining buffer until clear white bands appeared against a dark blue background. The white bands of MMP-2 and -9 were digitized and quantified using the ImageJ software (NIH Image, Bethesda, MD, USA).

### Statistical analysis

All data are expressed as the mean ± SD. Statistical comparisons between different patient groups were performed using Student’s *t*-test. Statistical significance was set at* p* < 0.05.

## Results

### Baseline characteristics

Aqueous humor samples from 10 patients with CRVO, and eight control subjects were included in the analysis. Aqueous humor samples from five patients with CRVO and four control subjects were used for the miRNA PCR array, and the remaining five CRVO and four control samples were used for gelatin zymography analysis. The baseline clinical characteristics of the enrolled subjects are shown in Table 1.

**Table 1:** Clinical characteristics and ophthalmic data of control subjects and patients with central retinal vein occlusion.

	M/F	Age (y)	Diagnosis	Laterality	Underlying diseases	CMT (μm)	BCVA (logMAR)	IOP (mmHg)	SE (D)	miRNA PCR array	Gel zymography
**Control**											
1	F	65	Cataract	OD	HTN	n/a	0.30	15	− 1.25	√	
2	F	63	Cataract	OD	(–)	n/a	0.52	19	− 0.25	√	
3	F	65	Cataract	OD	(–)	n/a	0.22	16	0.00	√	
4	F	62	Cataract	OD	(–)	n/a	0.00	17	− 0.50	√	
5	M	66	Cataract	OS	(–)	n/a	0.30	17	− 4.50		√
6	F	65	Cataract	OD	HTN	n/a	0.15	15	− 4.50		√
7	M	56	Cataract	OD	(–)	n/a	0.40	15	− 0.50		√
8	F	70	Cataract	OD	HTN	n/a	0.10	14	− 0.50		√
**CRVO**											
1	M	62	CRVO	OD	HTN, Dyslipidemia	603	0.52	12	0.50	√	
2	F	75	CRVO	OS	HTN, Unstable angina	567	0.30	13	− 0.25	√	
3	F	63	CRVO	OS	Dyslipidemia	802	1.00	18	1.00	√	
4	M	75	CRVO	OS	HTN	550	0.70	10	0.00	√	
5	M	52	CRVO	OD	HTN	657	1.10	15	0.00	√	
6	M	68	CRVO	OD	HTN	836	0.30	18	− 0.50		√
7	M	76	CRVO	OD	(–)	486	0.70	19	− 1.00		√
8	F	43	CRVO	OS	(–)	445	0.30	11	− 1.00		√
9	M	78	CRVO	OS	HTN	629	1.00	16	− 1.50		√
10	F	61	CRVO	OD	HTN, Dyslipidemia	391	1.00	12	0.75		√

BCVA, Best-corrected visual acuity; CMT, central macular thickness; CRVO, central retinal vein occlusion; HTN, hypertension; IOP, intraocular pressure; n/a, not applicable; SE, spherical equivalent.

### Analysis of the miRNA array in aqueous humor

**Figure 1:**
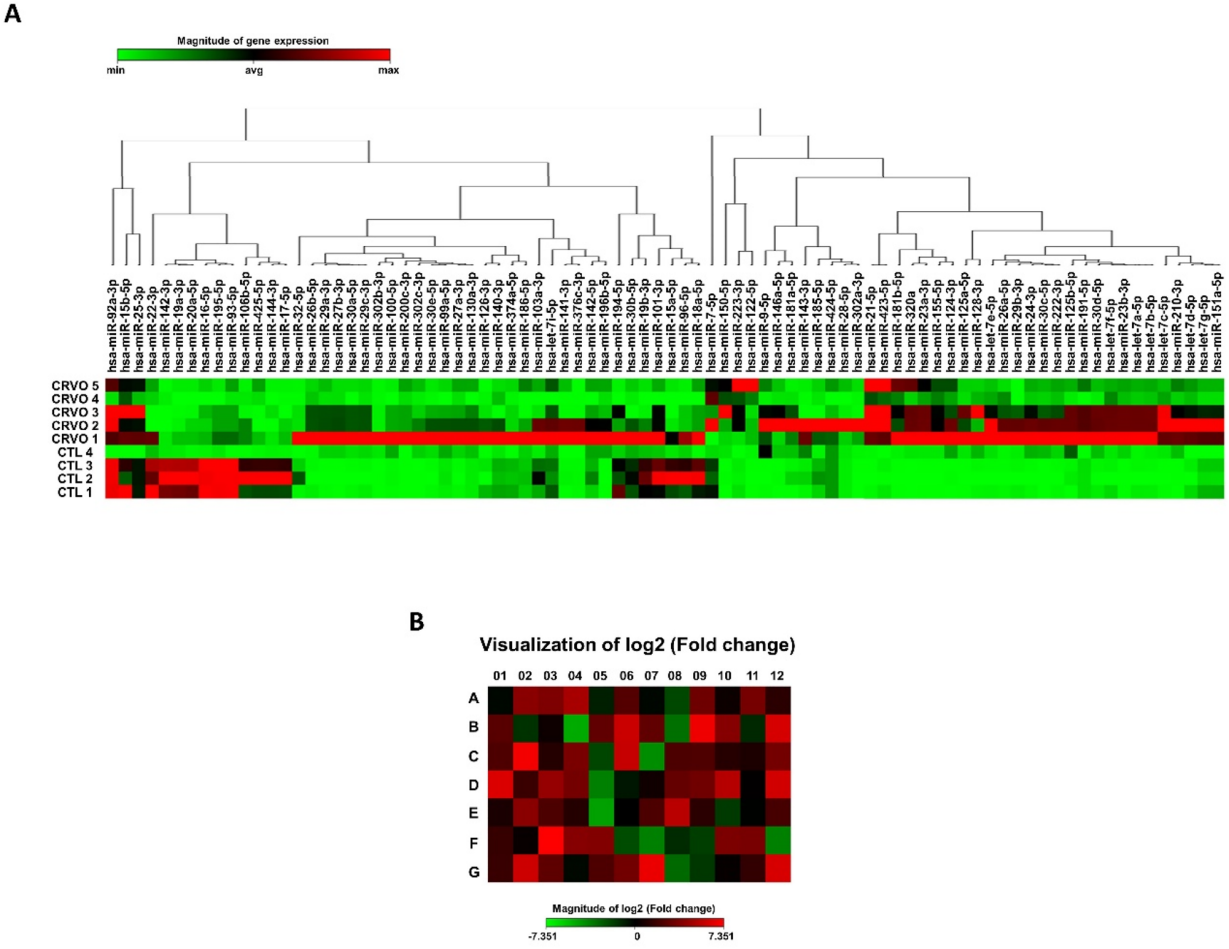
Clustergram and heatmap images representing miScript miRNA PCR array results using aqueous humor samples of patients with CRVO and control subjects. (**A**) Clustergram shows co-regulated genes across each sample. (**B**) Heatmap displays fold regulation expression data of 84 genes between the CRVO and control groups. The color reflects the magnitude of gene expression.

The miRNA array of 84 miRNAs showed 25 differentially expressed miRNAs in the CRVO group compared to the control (Fig. 1). The expression of eight miRNAs (*hsa-mir-16-5p, hsa-mir-142-3p, hsa-mir-19a-3p, hsa-mir-144-3p, hsa-mir-195-5p, hsa-mir-17-5p, hsa-mir-93-5p,* and *hsa-mir-20a-5p*) was significantly decreased and that of seventeen miRNAs (*hsa-let-7d-5p, hsa-miR-155-5p, hsa-miR-181b-5p, hsa-miR-21-5p, hsa-miR-223-3p, hsa-miR-210-3p, hsa-miR-320a, hsa-miR-423-5p, hsa-let-7a-5p, hsa-miR-124-3p, hsa-miR-23a-3p, hsa-let-7e-5p, hsa-miR-23b-3p, hsa-miR-191-5p, hsa-let-7b-5p, hsa-let-7c-5p,* and *hsa-let-7f.-5p*) was significantly increased in the AH of the patients with CRVO (Supplementary Figure S1).

### Bioinformatics

**Figure 2:**
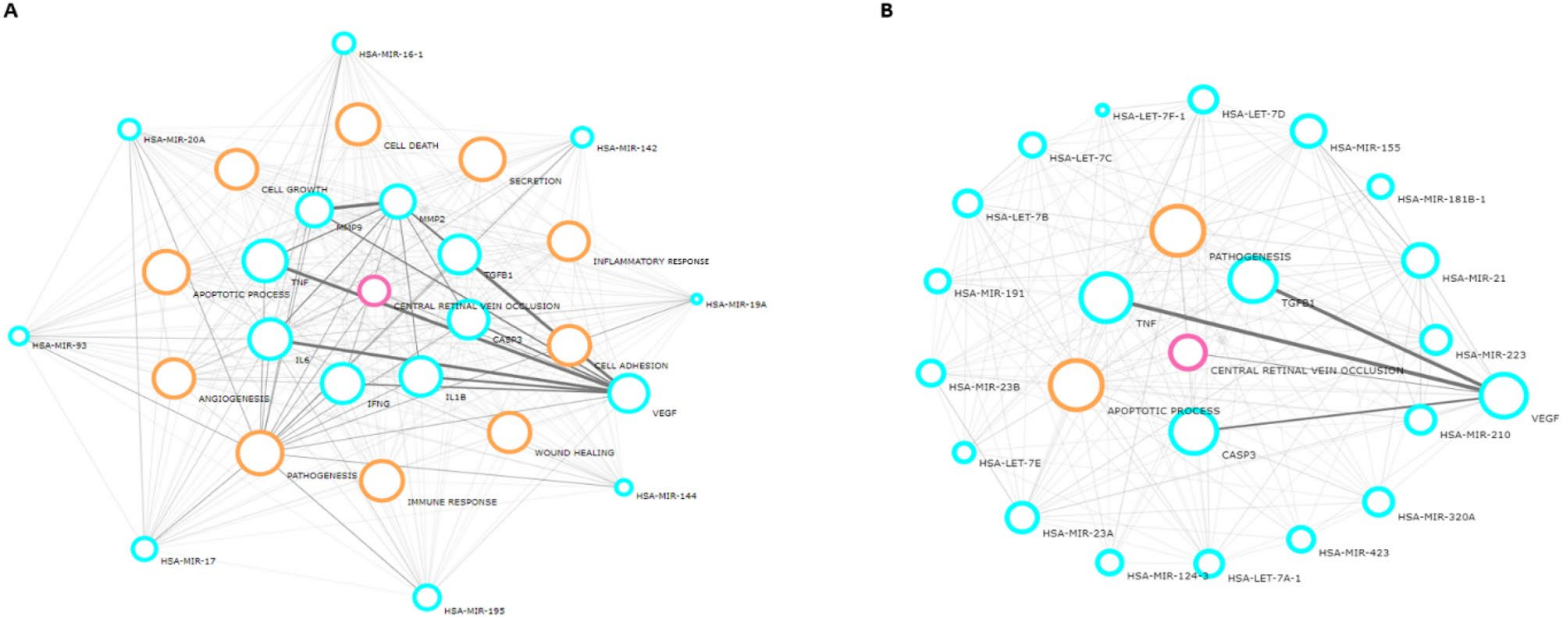
The relationship between miRNA genes and CRVO disease using a bioinformatics analysis. (**A**) Eight miRNAs (*hsa-mir-16-5p, hsa-mir-142-3p, hsa-mir-19a-3p, hsa-mir-144-3p, hsa-mir-195-5p, hsa-mir-17-5p, hsa-mir-93-5p,* and *hsa-mir-20a-5p*) were significantly downregulated in the CRVO group. Vascular endothelial growth factor (VEGF), matrix metalloproteinases-2 (MMP-2), MMP-9, tumor necrosis factor (TNF), transforming growth factor beta-1 (TGFB1), caspase-3 (CASP3), interleukin-6 (IL6), interferon gamma (IFNG), and interleukin-1-beta (IL1B) were related to the 8 miRNAs and CRVO disease. (**B**) Seventeen miRNAs (*hsa-let-7d-5p, hsa-miR-155-5p, hsa-miR-181b-5p, hsa-miR-21-5p, hsa-miR-223-3p, hsa-miR-210-3p, hsa-miR-320a, hsa-miR-423-5p, hsa-let-7a-5p, hsa-miR-124-3p, hsa-miR-23a-3p, hsa-let-7e-5p, hsa-miR-23b-3p, hsa-miR-191-5p, hsa-let-7b-5p, hsa-let-7c-5p,* and *hsa-let-7f.-5p*) were significantly upregulated in CRVO patients compared to control subjects. Four factors, VEGF, TNF, TGFB1, and CASP3, were associated with the 17 miRNAs and CRVO disease. Orange circles represent the related pathway between the 8 / 17 miRNAs and CRVO disease.

The eight downregulated miRNAs and seventeen upregulated miRNAs in the AH of the patients with CRVO were further analyzed using bioinformatic tools to identify the association with CRVO. Results revealed a direct network in the downregulated (Fig. 2A) and upregulated miRNA groups (Fig. 2B). In the direct network of downregulated miRNAs, MMP-2, MMP-9, tumor necrosis factor (TNF), transforming growth factor beta 1 (TGFB1), caspase 3 (CASP3), interleukin-6 (IL6), interferon gamma (IFNG), and interleukin-1-beta (IL1B) were directly related to these eight miRNAs and CRVO. All of these proteins were also related to VEGF. Pathway analysis revealed that inflammatory response, angiogenesis, pathogenesis, immune response, cell death, cell growth, cell adhesion, secretion, apoptotic process, and wound healing were directly related to these proteins and miRNAs. In the direct network of upregulated miRNAs, TNF, TGFB1, and CASP3, which are also related to VEGF, were shown to be directly related to these 17 miRNAs and CRVO. Further, pathway analysis also revealed that the apoptotic process and pathogenesis were directly associated with all these proteins and miRNAs. Finally, we selected MMP-2 and MMP-9 to investigate whether these MMPs differed in terms of activity in the AH of patients with CRVO compared with control subjects.

### MMP-2 and MMP-9 activity in the aqueous humor

**Figure 3:**
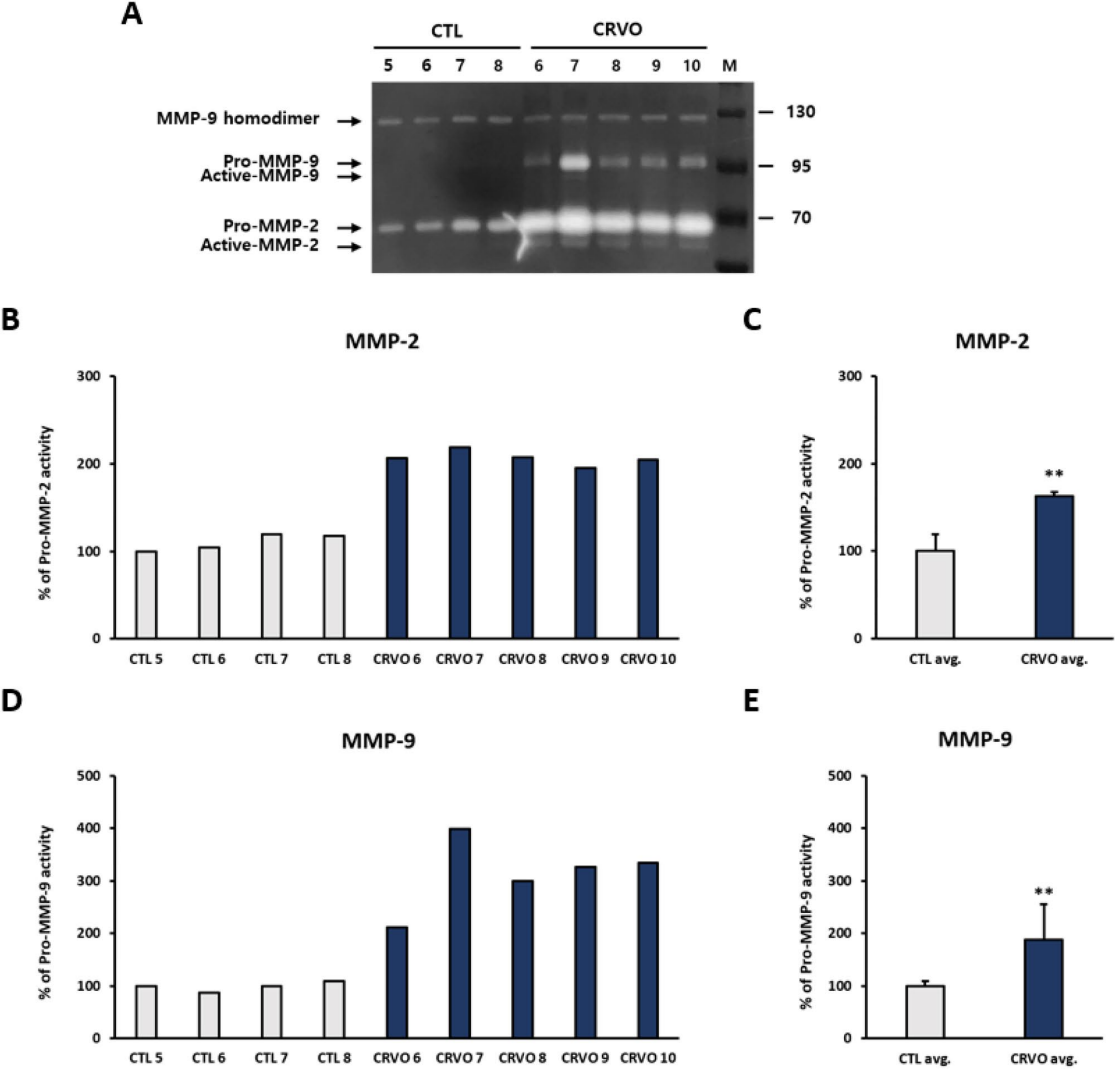
Gelatin zymography evaluating gelatinases activity in the aqueous humor of patients with CRVO and control subjects. (**A**) Acrylamide gel containing 0.1% gelatin was reacted with white bands by MMP-2 and -9 gelatinases. (**B**, **D**) % of pro-MMP-2 and MMP-9 activity are displayed in the graphs. (**C**, **E**) The averages of the control and CRVO groups were expressed as the % of pro-MMP-2 and -9 activity. Both results were increased in the CRVO group compared with the control group.

Gelatin zymography was performed to compare the activity of MMP-2 and -9 between CRVO and control groups, using the AH of five patients with CRVO and four control subjects. MMP-2 and MMP-9 activity was significantly increased in the AH of patients with CRVO (Fig. 3, *p* < 0.01).

## Discussion

**Figure 4:**
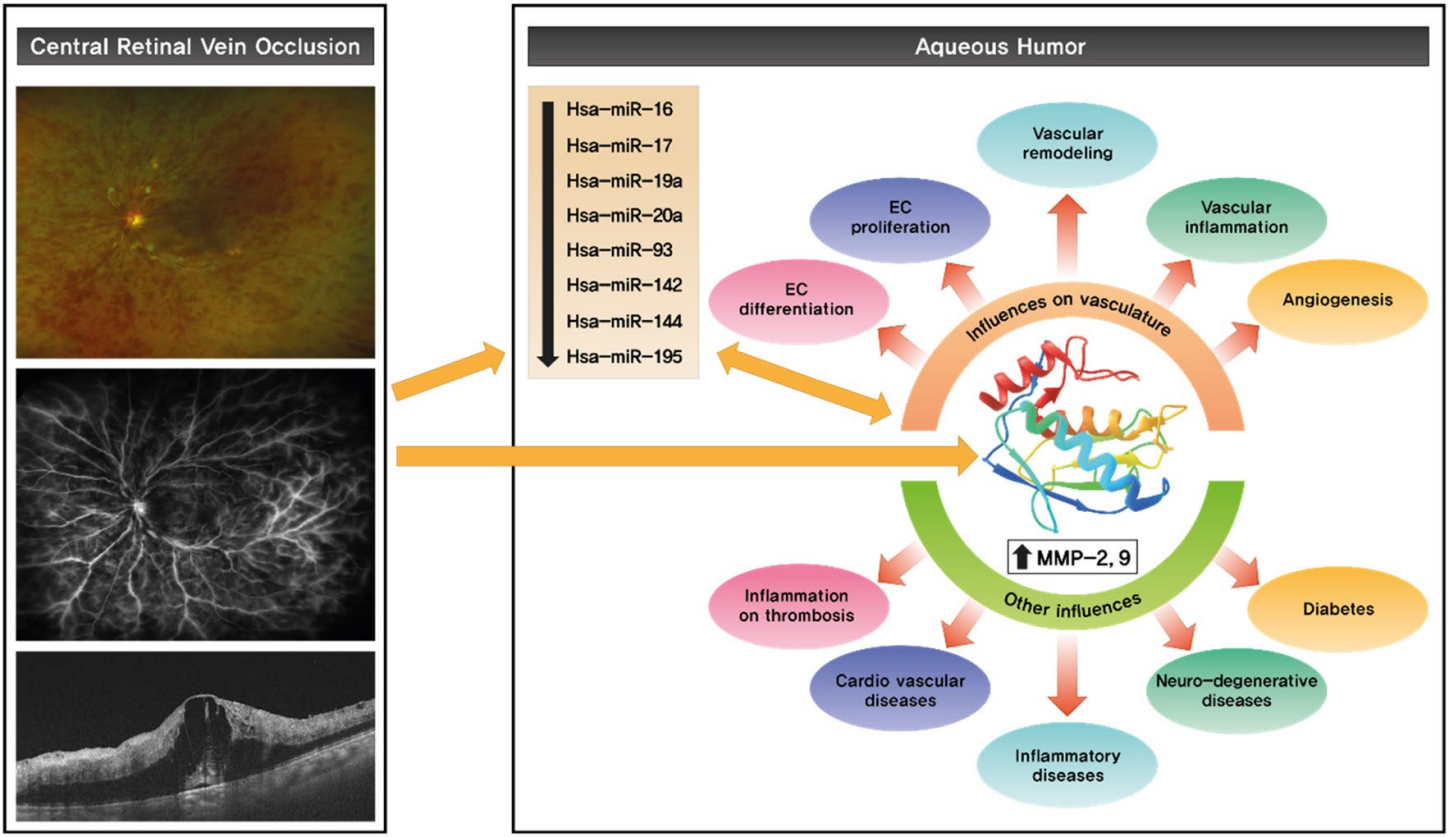
Schematic representation of the main findings. The left column shows the representative retinal images of patients with CRVO (no. 3. patients). Ultra-wide fundus photography (wFP) indicates diffuse retinal hemorrhage, cotton wool spots, and hard exudates. Fluorescein angiography (FA) indicates a diffuse nonperfusion area and vascular leakage. Optical coherent tomography (OCT) images indicate macular edema and ischemic retina. The right column indicates the aqueous humor findings, namely the decreased expression of miRNAs and increased activity of MMP-2 and -9, and the possible influences and roles of these MMPs.

In the present study, we found 8 downregulated and 17 upregulated miRNAs in the AH of treatment naïve patients with CRVO. Among these differentially expressed miRNAs, eight downregulated miRNAs were directly related to MMP-2 and -9 in CRVO. We also confirmed that MMP-2 and -9 activity was significantly increased in the AH of patients with CRVO. Therefore, the downregulation of these eight miRNAs may be related to the increased activity of MMP-2 and -9 in treatment naïve CRVO group. This is the first study to investigate the differential expression of miRNAs in the AH of patients with CRVO and the possible relationship between increased MMP-2 and -9 activity and downregulated miRNAs in CRVO. A schematic summary of the main findings of this study is shown in Fig. 4.

To the best of our knowledge, this is the first report on the expression and role of miRNAs in retinal vein occlusion (RVO). Retinal neovascularization is a common advanced stage of several retinal vascular diseases, including RVO and diabetic retinopathy. Several studies have highlighted the role of miRNAs in retinal neovascularization^20^. For example, decreased expression of miR-128 in oxygen-induced retinopathy mice and its role in inhibiting retinal neovascularization have been reported^21^. Additionally, miR-126 inhibits ischemia-induced retinal neovascularization by regulating the expression of angiogenic growth factors^22^, and miR-410 inhibits retinal neovascularization in an oxygen-induced retinopathy mouse model^23^. However, the AH miRNA levels in patients with CRVO have not yet been reported. Herein, we used AH samples from treatment naïve patients with CRVO and none of the patients developed retinal neovascularization.

Bioinformatic analysis of these downregulated miRNAs in CRVO showed a direct relationship with VEGF, MMP-2 and -9, TNF, TGFB1, CASP3, IL6, IFNG, and IL1B. The roles of angiogenic and proinflammatory factors, including VEGF, TNF, TGFB1, CASP3, IL6, IFNG, and IL1B, and their association with miRNAs and the specified proteins in retinal diseases have been previously reported^20,22–28^. MMP-2 and -9 are migration- and invasion-related proteins, which play a critical role in cell invasion by stimulating the degradation of the ECM and cell migration. Additionally, miRNAs that regulate MMPs in various diseases, including tumors, were previously reported^29^. However, the role of miRNAs in regulating MMPs in retinal diseases is rarely reported. Although only one recent study has reported that miR-22 targets MMP in diabetic animal models^30^, the relationship between miRNAs and MMP in retinal ischemia remains unclear. Therefore, among the related proteins identified by bioinformatics analysis, MMP-2 and -9 were considered relatively novel factors with little research done on CRVO (especially in relation to miRNA), thus we decided to target AH MMP-2 and -9 in CRVO as the next step in this study.

The downregulated miRNAs were *hsa-mir-16*, *hsa-mir-142*, *hsa-mir-19a*, *hsa-mir-144*, *hsa-mir-195*, *hsa-mir-17*, *hsa-mir-93*, and *hsa-mir-20a*. Overexpression of *miR-16* reduced the expression levels of MMP-2 and -9 in cancer cells, thus inhibiting cell invasion and migration^31^, which was suggested to be due to the inhibition of the Bcl-2 and nuclear factor-κB1/MMP-9 pathway^32^. MMP-2 and -9 expression levels were reduced in *miR-142* overexpressed cancer cells to inhibit migration and invasion of these cells^33^. Additionally, *miR-142* seemingly acts as a tumor suppressor by reducing the expression of MMP-2 and -9, thereby inhibiting tumor cell invasion^34^. MMP-2 and -9 expression is downregulated by *miR-19a* to suppress the invasion of fibroblasts, resulting in the inhibition of fibrogenesis^35^. MMP-2 and -9 expression was reduced by *miR-144* overexpression and was increased by *miR-144* inhibition, and E26 transformation specific-1 (ETS-1), the transcriptional oncoprotein, was thought to be a molecular target of *miR-144*^36^. ETS-1 is known as a mediator of proinflammatory responses in endovascular injury by regulating the activation of proinflammatory cytokines and adhesion molecules, including IL-6, monocyte chemotactic protein (MCP)-1, P-selectin, and E-selectin^37^. Additionally, *miR-195* downregulated MMP-2 and -9 and was expected to suppress the invasion of cancer cells by targeting DCN1-like protein 1, which is known as squamous cell carcinoma-related oncogene^38^. Decreased MMP-2 expression resulting from the upregulation of *miR-93* was reported, possibly also inhibiting the proliferation and metastasis of tumor cells^39^. Upregulation of *miR-20a* inhibited the expression of MMP-2 and -9 in inflammatory diseases, thereby regulating disease progression^40^. Overall, it has been found in previous studies on diseases other than ophthalmic diseases (mostly in cancer) that these miRNAs (*hsa-mir-16*, *hsa-mir-142*, *hsa-mir-19a*, *hsa-mir-144*, *hsa-mir-195*, *hsa-mir-93*, and *hsa-mir-20a*) play a role in inhibiting cell migration and invasion by regulating MMP-2 or -9. Further research is needed on the direct mechanism and role of these miRNAs and MMP-2 and -9 in CRVO. In contrast, the effects of *miR-17* differed from what was expected. Numerous studies have reported that *miR-17* inhibition reduces MMP-2 and -9 activity^41^. The relationship between *miR-17* and MMPs may differ in CRVO. Therefore, the role of *miR-17* and its effects on MMPs should be investigated in future studies.

The role of MMPs in systemic vascular diseases is well reported. Increased serum MMP-2 and -9 level correlates with the severity and progression of cerebral infarctions^42,43^. In the peripheral vasculature, upregulated MMP expression in the lower extremities, caused by an increase in hydrostatic venous pressure, could lead to degradation of ECM, dysfunction of vasoconstriction and relaxation, and ultimately lead to venous thrombosis and thrombophlebitis^44^. Although limited, abnormal MMP expression has also been reported in retinal vascular diseases, such as diabetic retinopathy and RVO^16,18,45^. These studies suggested that activation of intraocular MMPs (-1, -2, -9) affects the stability and elasticity of retinal vasculature^16^. A previous study reporting the effect of an antioxidant with respect to decreasing the MMP-9 levels and resolving retinal edema in a murine RVO model, suggested that the partial mechanism of retinal hyperpermeability in RVO is associated with MMP-9 production^46^. In the current study, bioinformatic analysis showed a direct relationship between MMP-2/-9 and various pathways (inflammatory response, angiogenesis, pathogenesis, immune response, cell death, cell growth, cell adhesion, secretion, apoptotic process, and wound healing) in CRVO. Therefore, although the exact mechanism remains to be elucidated, increased MMP-2 and -9 expression affects various inflammatory processes, leading to macular edema which can cause severe visual impairment. This suggests that the regulation of MMPs may play a potential role in disease progression or treatment of CRVO.

Serum or intraocular MMP levels in patients with RVO have been previously reported in a few studies^16–18,47^. Higher serum MMP-2 and -9 levels have been reported in patients with RVO compared to controls^47^. However, serum samples mainly reflect systemic status and are limited in that they do not directly reflect the intraocular status caused by CRVO. One of the two studies on vitreous samples showed higher intravitreal MMP-9 levels in RVO. However, the intravitreal MMP-2 levels between the RVO and control groups did not differ significantly^18^. Another study using vitreous samples reported higher intravitreal and serum MMP-9 levels in patients with CRVO than in control subjects^17^. Vitreous humor samples accurately reflect retinal conditions, but the sample collection process is more invasive and is considered less clinically applicable than AH samples. One previous study using AH samples reported significantly higher MMP-1, -2, -7, and -9 levels in patients with CRVO than in control subjects^16^, supporting our findings. The authors suggested that activation of these MMPs would affect the stability and elasticity of the retinal vasculature and may directly contribute to CRVO pathogenesis^16^. The difference compared with our study was that it used a Luminex multiplex assay to quantify the level of aqueous humor MMPs. Secreted MMPs are produced in inactive forms and must be activated extracellularly to perform their functions, which is then regulated by protease inhibitors. Therefore, activity assays may be more representative of the role of MMPs in pathogenic states than protein production analysis^48^. Thus, we quantitatively measured MMP activity using zymography in the current study. Zymography has been used to assess MMP-2 and -9 activity and is more sensitive than western blotting for quantifying MMPs^49,50^. The analysis of AH MMPs activity using zymography was conducted in this study for the first time. Furthermore, no previous studies have mentioned a link between MMPs and miRNAs expressed differently in CRVO. We analyzed miRNAs that are differentially expressed in AH of CRVO and found from bioinformatics results that they are associated with MMP-2 and -9, and revealed that MMP-2 and -9 also exhibited differential activities in the AH of CRVO using zymography analysis for the first time, which is considered to be the strength of this study.

Our study is not without limitations. First, a relatively small number of participants were included. Second, the amount of AH was not sufficient to simultaneously analyze both miRNAs and MMPs at once and had to be investigated separately using other samples. Thus, no direct comparisons could be made between the miRNA and MMP levels. Further in vitro and animal studies are needed to determine the role of miRNAs and MMPs in CRVO. Nevertheless, the strength of the present study is that we used AH, which is easier to approach and safer to obtain than vitreous samples, and is more representative of the retinal disease condition than serum samples. Besides, we analyzed MMP activity using gelatin zymography. This is the first study to investigate the differential expression of aqueous humor miRNAs and the potential relationship with MMPs in patients with CRVO. Further studies are needed to elucidate whether these miRNAs control MMP-2 and -9 in CRVO, and whether MMP-2 and -9 regulation via associated miRNAs could be targeted as an alternative or adjunct treatment in CRVO. In addition, we identified 17 upregulated miRNAs in the AH of CRVO patients, which showed a direct relationship with TNF, TGFB1, CASP3, and VEGF. As described, we focused on MMP-2 and -9 in the current study, as this was a novel finding. However, the upregulated miRNAs discovered in the AH of patients with CRVO were also described for the first time in this study. Thus, further studies on these upregulated miRNAs may help reveal the mechanism of pathogenesis of CRVO.

In conclusion, our results showed differentially expressed aqueous humor miRNAs and suggested a possible relationship between downregulated miRNAs and increased MMP activity in patients with CRVO. Downregulated miRNAs likely play a role in the regulation of MMP-2 and -9 activity in CRVO pathogenesis and progression. However, the exact mechanism by which these miRNAs are downregulated and subsequently regulate MMPs in CRVO remains to be elucidated. Notably, it may be possible to utilize aqueous humor MMP-2 and -9 as either novel biomarkers to evaluate disease state and treatment effect, or as therapeutic targets in CRVO.

## Supplementary Information

Supplementary Information.

## Data Availability

The data that support the findings of this study are available from the corresponding author upon reasonable request.
