# Supplementary material for: Differential expression of aqueous humor microRNAs in central retinal vein occlusion and its association with matrix metalloproteinases: a pilot study
Source: Sci Rep. 2022 Sep 30;12:16429. doi: 10.1038/s41598-022-20834-z (PMC9525721; doi:10.1038/s41598-022-20834-z)

Supplementary Figure S1. ***P*-value and fold regulation data of the expression of 84 miRNAs in the aqueous humor of patients with central retinal vein occlusion (CRVO), compared with control subjects.**

Of the 25 miRNAs with significant *p*-values, 17 showed increased fold regulation and 8 showed decreased fold regulation.

miRNA	p-value	Fold regulation	miRNA	p-value	Fold regulation
hsa-let-7d-5p	0.037	5.33	hsa-miR-30b-5p	0.821	-2.21
hsa-miR-155-5p	0.011	56.83	hsa-miR-30e-5p	0.205	4.67
hsa-miR-181b-5p	0.008	122.79	hsa-miR-200c-3p	0.149	131.33
hsa-miR-21-5p	0.001	71.16	hsa-miR-15b-5p	0.619	2.02
hsa-miR-223-3p	0.037	11.37	hsa-miR-194-5p	0.793	-4.04
hsa-miR-210-3p	0.037	53.06	hsa-miR-15a-5p	0.109	-17.41
hsa-miR-320a	0.008	10.20	hsa-miR-181a-5p	0.209	4.55
hsa-miR-423-5p	0.002	7.46	hsa-miR-125b-5p	0.094	4.81
hsa-let-7a-5p	0.036	9.23	hsa-miR-99a-5p	0.271	2.15
hsa-miR-124-3p	0.030	37.28	hsa-miR-28-5p	0.349	1.75
hsa-miR-23a-3p	0.006	65.89	hsa-miR-125a-5p	0.075	81.77
hsa-let-7e-5p	0.046	16.01	hsa-miR-29b-3p	0.129	3.22
hsa-miR-23b-3p	0.031	41.77	hsa-miR-29a-3p	0.106	20.09
hsa-miR-191-5p	0.047	2.93	hsa-miR-141-3p	0.165	10.17
hsa-let-7b-5p	0.032	13.48	hsa-miR-18a-5p	0.443	-1.56
hsa-let-7c-5p	0.031	61.50	hsa-miR-374a-5p	0.522	1.39
hsa-let-7f-5p	0.023	9.82	hsa-miR-92a-3p	0.851	1.05
hsa-miR-16-5p	0.018	-4.29	hsa-miR-25-3p	0.322	1.70
hsa-miR-142-3p	0.011	-31.49	hsa-miR-376c-3p	0.208	4.63
hsa-miR-19a-3p	0.014	-12.92	hsa-miR-126-3p	0.321	2.08
hsa-miR-144-3p	0.036	-25.78	hsa-miR-424-5p	0.542	1.00
hsa-miR-195-5p	0.025	-3.13	hsa-miR-30a-5p	0.150	4.48
hsa-miR-17-5p	0.048	-12.86	hsa-miR-151a-5p	0.093	2.35
hsa-miR-93-5p	0.037	-2.32	hsa-miR-143-3p	0.334	-1.06
hsa-miR-20a-5p	0.018	-8.16	hsa-miR-30d-5p	0.063	4.11
hsa-miR-142-5p	0.339	-1.11	hsa-let-7i-5p	0.285	1.19
hsa-miR-9-5p	0.212	15.50	hsa-miR-302a-3p	0.286	163.20
hsa-miR-150-5p	0.065	12.33	hsa-miR-222-3p	0.053	14.60
hsa-miR-27b-3p	0.123	29.22	hsa-miR-19b-3p	0.403	-4.57
hsa-miR-101-3p	0.592	-1.82	hsa-miR-186-5p	0.410	-3.47
hsa-miR-103a-3p	0.577	-1.12	hsa-miR-196b-5p	0.153	12.08
hsa-miR-26a-5p	0.051	9.11	hsa-miR-27a-3p	0.137	11.06
hsa-miR-32-5p	0.629	1.27	hsa-miR-22-3p	0.073	-12.76
hsa-miR-26b-5p	0.099	10.70	hsa-miR-130a-3p	0.173	3.10
hsa-let-7g-5p	0.093	2.36	hsa-miR-29c-3p	0.133	6.58
hsa-miR-30c-5p	0.078	5.72	hsa-miR-140-3p	0.402	-1.19
hsa-miR-96-5p	0.277	-2.75	hsa-miR-128-3p	0.123	4.69
hsa-miR-185-5p	0.430	1.32	hsa-miR-122-5p	0.163	110.05
hsa-miR-24-3p	0.056	6.93	hsa-miR-106b-5p	0.098	-2.90
hsa-miR-146a-5p	0.174	6.82	hsa-miR-7-5p	0.467	1.14
hsa-miR-425-5p	0.060	-9.35	hsa-miR-100-5p	0.331	2.75
hsa-miR-302b-3p	0.393	14.33	hsa-miR-302c-3p	0.217	76.11

Supplementary Figure S2. Original gelatin zymography gel images of Figure 3.

The original uncropped gelatin zymography gel image of Figure 3 was shown in Supplementary Figure S2. Marker sizes were indicated on the right.

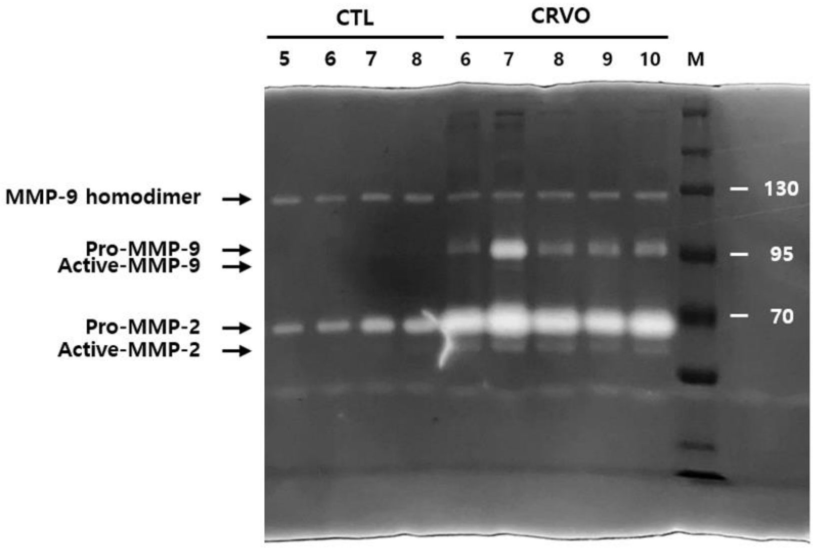

Supplement: Supplementary file 1 — Supplementary Information. [file 41598_2022_20834_MOESM1_ESM.pdf]
